# Noninvasive hematocrit assessment for cardiovascular magnetic resonance extracellular volume quantification using a point-of-care device and synthetic derivation

**DOI:** 10.1186/s12968-018-0443-1

**Published:** 2018-03-15

**Authors:** Sean Robison, Gauri Rani Karur, Rachel M. Wald, Paaladinesh Thavendiranathan, Andrew M. Crean, Kate Hanneman

**Affiliations:** 1 Department of Medical Imaging, Toronto General Hospital, University Health Network, University of Toronto, 585 University Ave, 1PMB-298, Toronto, ON M5G 2N2 Canada; 2 Division of Cardiology, Department of Medicine, Peter Munk Cardiac Center, Toronto General Hospital, University of Toronto, Toronto, Canada

**Keywords:** Cardiovascular magnetic resonance (CMR), T1 mapping, Extracellular volume (ECV), Noninvasive hemoglobin monitoring, Hematocrit

## Abstract

**Background:**

Calculation of cardiovascular magnetic resonance (CMR) extracellular volume (ECV) requires input of hematocrit, which may not be readily available. The purpose of this study was to evaluate the diagnostic accuracy of ECV calculated using various noninvasive measures of hematocrit compared to ECV calculated with input of laboratory hematocrit as the reference standard.

**Methods:**

One hundred twenty three subjects (47.7 ± 14.1 years; 42% male) were prospectively recruited for CMR T1 mapping between August 2016 and April 2017. Laboratory hematocrit was assessed by venipuncture. Noninvasive hematocrit was assessed with a point-of-care (POC) device (Pronto-7^®^ Pulse CO-Oximeter^®^, Masimo Personal Health, Irvine, California, USA) and by synthetic derivation based on the relationship with blood pool T1 values. Left ventricular ECV was calculated with input of laboratory hematocrit (Lab-ECV), POC hematocrit (POC-ECV), and synthetic hematocrit (synthetic-ECV), respectively. Statistical analysis included Wilcoxon signed-rank test, Bland-Altman analysis, receiver-operating curve analysis and intra-class correlation (ICC).

**Results:**

There was no significant difference between Lab-ECV and POC-ECV (27.1 ± 4.7% vs. 27.3 ± 4.8%, *p* = 0.106), with minimal bias and modest precision (bias − 0.18%, 95%CI [− 2.85, 2.49]). There was no significant difference between Lab-ECV and synthetic-ECV (26.7 ± 4.4% vs. 26.5 ± 4.3%, *p* = 0.084) in subjects imaged at 1.5 T, although bias was slightly higher and limits of agreement were wider (bias 0.23%, 95%CI [− 2.82, 3.27]). For discrimination of abnormal Lab-ECV ≥30%, POC-ECV had good diagnostic performance (sensitivity 85%, specificity 96%, accuracy 94%, and AUC 0.902) and synthetic-ECV had moderate diagnostic performance (sensitivity 71%, specificity 98%, accuracy 93%, and AUC 0.849). POC-ECV had excellent test-retest (ICC 0.994, 95%CI[0.987, 0.997]) and inter-observer agreement (ICC 0.974, 95%CI[0.929, 0.991]).

**Conclusions:**

Myocardial ECV can be accurately and reproducibly calculated with input of hematocrit measured using a noninvasive POC device, potentially overcoming an important barrier to implementation of ECV. Further evaluation of synthetic ECV is required prior to clinical implementation.

## Background

Myocardial extracellular volume (ECV) fraction derived from cardiovascular magnetic resonance (CMR) T1 mapping has been validated based on histology [1–3], with demonstrated prognostic significance in several conditions [4–6]. Calculation of ECV requires accurate and timely assessment of hematocrit, to correct for the blood contrast volume of distribution [7]. Traditional laboratory determination of hematocrit via venipuncture in the setting of ECV evaluation is costly, mildly uncomfortable for the patient, and often inconvenient. Therefore, rapid noninvasive determination of hematocrit would be beneficial in the setting of ECV analysis.

Noninvasive point-of-care (POC) devices have recently become commercially available, providing immediate hemoglobin and hematocrit results without the need for blood sampling by venipuncture or finger prick [8–10]. Additionally, synthetic derivation of hematocrit has recently been described based on the relationship between hematocrit and non-contrast blood pool T1 values [11]. However, there are important limitations to synthetic analysis, as each specific acquisition scheme requires derivation and validation [12]. A recent study suggests that synthetic ECV may result in miscategorization of individual patients [13].

The purpose of this study was to evaluate the diagnostic accuracy of CMR ECV calculated using noninvasive measures of hematocrit (determined with a POC device and synthetic derivation) compared to ECV calculated with input of laboratory hematocrit as the reference standard. We hypothesized that there would be no significant difference in myocardial ECV calculated using noninvasive measures of hematocrit as compared with ECV derived from laboratory hematocrit.

## Methods

### Study population

Institutional research ethics board approval was obtained for this prospective study. Between August 2016 and April 2017, 149 subjects undergoing clinical or research CMR were prospectively recruited for T1 mapping with calculation of ECV. Written informed consent was obtained from all study subjects. Exclusion criteria included incomplete or aborted CMR (*n* = 4), subject refusal of intravenous contrast administration (*n* = 2), substantial artifact precluding image analysis (*n* = 4), and failure of POC device analysis (due to the presence of gel nail polish, marked hand tremor, and/or inadequate signal) (*n* = 16). The final cohort of 123 subjects included both patients and healthy subjects (47.7 ± 14.1 years; 42% male). Clinical and demographic information were extracted from the electronic patient record.

### CMR technique

CMR was performed at 1.5 T (*n* = 74, Magnetom Avanto, Siemens Healthineers, Erlangen, Germany) or 3 T (*n* = 34, Magnetom Skyra, Siemens Healthineers; and *n* = 15 Biograph mMR, Siemens Healthineers). Retrospectively gated balanced steady-state free precession (bSSFP) cine images were obtained for assessment of left ventricular (LV) size and function by a stack of short-axis slices with coverage from cardiac base to the apex with 8-mm thickness and 2-mm inter-slice gap. T1 mapping was performed using a bSSFP readout modified Look-Locker inversion recovery (MOLLI) acquisition scheme with three short-axis slices (8 mm slice thickness) acquired at basal, mid and apical locations, pre-contrast and 12–15 min after administration of 0.15 mmol/kg body weight of gadobuterol (Gadovist; Bayer Healthcare, Berlin, Germany) [14]. Multi-plane late gadolinium enhanced (LGE) imaging was performed approximately 15 min following contrast administration employing a 2D inversion recovery gradient-recalled echo sequence (slice thickness 8 mm and 2 mm inter-slice gap).

### CMR analysis

All studies were analyzed by a fellowship trained radiologist (SR, with 3 years CMR experience) blinded to all identifying clinical and imaging data. Evaluation of LV volumes and function was performed using commercially available software (QMASS MRI, Medis Medical Imaging Systems, Leiden, The Netherlands).

**Fig. 1:**
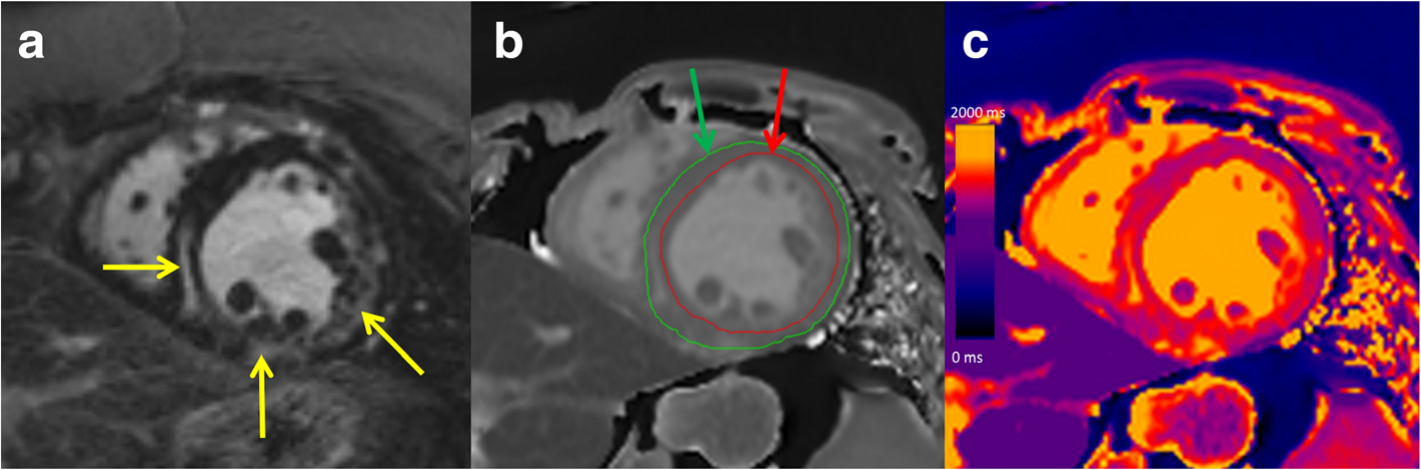
Example short axis late gadolinium enhanced (LGE) image (**a**), non-contrast T1 map with contours (**b**), and color non-contrast T1 map (**c**) in a 30-year-old female with cardiac sarcoidosis. Yellow arrows indicate the presence of LGE. Endocardial (red arrow) and epicardial (green arrow) contours are shown

After inline, non-rigid motion correction of individual T1 mapping images, an inline T1 map was generated using standard 3-parameter fitting. Analysis was performed off-line using commercially available software (cvi42; Circle Cardiovascular Imaging, Calgary, Alberta, Canada). Regions of interest were drawn manually in the LV blood pool on pre- and post-contrast T1 maps with care taken to avoid the myocardium and papillary muscles. Myocardial T1 values were calculated by contouring epicardial and endocardial borders at basal, mid and apical slices, with care taken to avoid blood pool and epicardial fat, Fig. 1. Myocardial segments with LGE were not excluded from T1 analysis. Blood pool and myocardial T1 values were averaged across the three slices.

### Hematocrit assessment

A small blood sample (< 30 mL) was collected by venipuncture within 24 h of CMR for the purpose of central hospital laboratory determination of hemoglobin and hematocrit (Lab-Hct). The time between laboratory blood sampling and CMR start time was recorded. Samples were analyzed by a hematology analyzer (CELL-DYN, Sapphire, Abbott Core Laboratory, Abbott Park, Illinois, USA), with reported bias and standard deviation of − 0.07 ± 1.17 g/dL [15]. Laboratory hemoglobin and hematocrit results were recorded blinded to clinical information and the results of POC and synthetic derivation analysis. Anemia was defined as a laboratory hemoglobin level < 122 g/L for females, < 132 g/L for males ≥60 years of age, and < 137 g/L for males < 60 years of age [16].

POC analysis was performed within 1 h of CMR using a Pronto-7^®^ Pulse CO-Oximeter (Masimo Personal Health, Irvine, California, USA), by an experienced observer (SR) blinded to all identifying information and the results of laboratory analysis and synthetic derivation. The POC probe was applied to the fingertip of the ring or middle finger according to the manufacturer’s guidelines. If the first attempt was unsuccessful with no result displayed, up to four additional attempts at POC analysis were performed. The total number of POC analysis attempts was recorded for each subject. The displayed hemoglobin value was recorded with subsequent derivation of hematocrit (POC-Hct) using a conversion of 0.3, according to previously published guidelines [17]. In a subset of subjects (*n* = 32), the total time for POC device analysis was determined, timed from turning the device on to recording the displayed hemoglobin value.

In the subgroup of subjects scanned at 1.5 T, synthetic hematocrit (synthetic-Hct) was calculated from the relationship between hematocrit and non-contrast blood pool T1 values as described previously, using a published linear regression formula for MOLLI acquisition schemes at 1.5 T [11]:

$$ \mathrm{Synthetic}-\mathrm{Hct}=\left(866.0\ast \left[1/T{1}_{\mathrm{blood}}\right]\right)-0.1232 $$

Synthetic hematocrit was calculated by an experienced observer (SR) who was blinded to clinical information and the results of POC and laboratory analysis. Synthetic-hematocrit was not evaluated in subjects imaged at 3 T due to lack of previously published formulas using the same CMR scanner that was used in this study. We did not fit a linear regression equation to our data given the relatively small number of subjects imaged at each field strength and acquisition scheme.

### ECV derivation

Myocardial ECV was calculated based on pre- and post-contrast myocardial and blood pool T1 values and hematocrit, as proposed by Arheden et al. [18]. ECV was calculated with input of Lab-Hct (Lab-ECV), POC-Hct (POC-ECV), and synthetic-Hct (synthetic-ECV), respectively. The same myocardial and blood pool T1 values were used in each ECV calculation.

### Test-retest and inter-observer agreement

To evaluate test-retest variability of POC measures, three POC analyses were performed in immediate succession in a subset of subjects (*n* = 42) without removal of the probe. In these subjects, the first result obtained was used in the general analysis.

To assess inter-observer agreement of POC measures, a second POC analysis was performed in a subset of subjects (*n* = 16) by a second experienced CMR fellowship trained reader (GK) independent from the first analysis, blinded to all identifying clinical data and the first set of measurements.

### Comparison of POC devices

Evaluation of hematocrit using a second POC device (Pronto^®^ Pulse CO-Oximeter, Masimo Personal Health) was performed in a group of 10 subjects. These 10 subjects also underwent parallel assessment using the Pronto-7^®^ POC device and central hospital laboratory determination. Myocardial ECV was calculated in this group with input of hematocrit from the Pronto^®^ device, Pronto-7^®^ device, and laboratory determination.

### Statistical analysis

Statistical analysis was performed using STATA v14.1 (Stata Corporation, College Station, Texas, USA). The sample size was calculated to detect a difference in ECV measurements between techniques of 1% with a standard deviation of ECV measurements of 3% using a paired t-test [19]. To detect this difference with a power of 95% and alpha error of 0.05, a total of 119 subjects were needed. All continuous data were first tested for normal distribution using the Shapiro-Wilk test. Continuous variables are described using mean and standard deviation or median and interquartile range (IQR), and categorical variables using numbers and percentage. Comparisons between values were made by paired t-test for continuous values with normal distribution and Wilcoxon signed-rank test for continuous values with non-normal distribution. Correlations between continuous variables were assessed with Pearson or Spearman correlation coefficient, as appropriate. Bias and precision were evaluated using Bland-Altman analysis. Test-retest and inter-observer agreement were assessed via the intra-class correlation coefficient (ICC) with two-way random effects model. Sensitivity analysis was performed, restricting the analysis to subjects in different subgroups including those with anemia, different field strengths (1.5 T and 3 T), gender (males and females) and clinical status (healthy subjects and patients). Diagnostic test performance of POC-ECV and synthetic-ECV in comparison to Lab-ECV as the reference standard was assessed using a Lab-ECV cut-off value of ≥30% as abnormal, including sensitivity, specificity, accuracy and area under the receiver operating curve (ROC) [20, 21]. A two-tailed *p*-value of < 0.05 was considered statistically significant.

## Results

Baseline characteristics and clinical details are summarized in Table 1. CMR findings are summarized in Table 2.

**Table 1:** Baseline Characteristics

Characteristic	Subjects (*n* = 123)
Age (years)	47.7 ± 14.1
Male	52 (42%)
BSA (m2)	1.9 ± 0.3
Heart rate (bpm)	69 ± 12
Indication for CMR	
Chemotoxicity	34 (28%)
Hypertrophic cardiomyopathy	24 (20%)
Anderson-Fabry disease	11 (9%)
Myocarditis/pericarditis	10 (8%)
Sarcoid	8 (7%)
Cardiomyopathy, cause unspecified	17 (14%)
Healthy subjects	19 (15%)
Field Strength	
1.5 T	74 (60%)
3 T	49 (40%)

Data are mean ± standard deviation or number of patients with percentage in parentheses

*BPM* beats per minute, *BSA* body surface area, *CMR* cardiovascular magnetic resonance

**Table 2:** Hemoglobin, Hematocrit and CMR Results

Measurement	Subjects (*n* = 123)
CMR Values	
LVEDV (mL)	152 (IQR 126–184)
Indexed LVEDV (mL/m^2^)	80 (IQR 71–96)
LVESV (mL)	64 (IQR 50–80)
Indexed LVESV (mL/m^2^)	34 (IQR 27–43)
LVSV (mL)	82 (IQR 69–103)
LVEF (%)	58 (IQR 52–64)
Laboratory Values	
Laboratory hemoglobin (g/L)	134.1 ± 17.0
Laboratory hematocrit	0.399 ± 0.048
Laboratory ECV (%)	27.1 ± 4.7
POC Values	
POC hemoglobin (g/L)	131.6 ± 14.7
POC hematocrit	0.395 ± 0.044
POC ECV (%)	27.3 ± 4.8
Synthetic Values	
Synthetic hematocrit	0.398 ± 0.031
Synthetic ECV (%)	26.5 ± 4.3
Elevated Laboratory ECV (≥30%)	31 (25%)
Anemia	31 (25%)

Data are mean ± standard deviation, median and interquartile range (IQR), or number of patients with percentage in parentheses

*CMR* cardiovascular magnetic resonance, *ECV* extracellular volume, *LVEDV* left ventricular end diastolic volume, *LVEF* left ventricular ejection fraction, *LVESV* left ventricular end systolic volume, *LVSV* left ventricular stroke volume, *POC* point-of-care

### Hematocrit assessment and ECV derivation

Hemoglobin, hematocrit and ECV values are detailed in Table 2. The median interval between blood sampling and CMR was 1.3 h (IQR 1.0–2.8 h). Mean laboratory hemoglobin and Lab-Hct values were 134.1 ± 17.0 g/L (range 79-169 g/L) and 0.399 ± 0.048 (range 0.239–0.509), respectively. The mean ratio of Lab-Hct to laboratory hemoglobin was 0.30 ± 0.01. A minority of subjects met criteria for anemia (*n* = 31, 25%). Mean Lab-ECV was 27.1 ± 4.7% (range 19.4–44.9%).

**Fig. 2:**
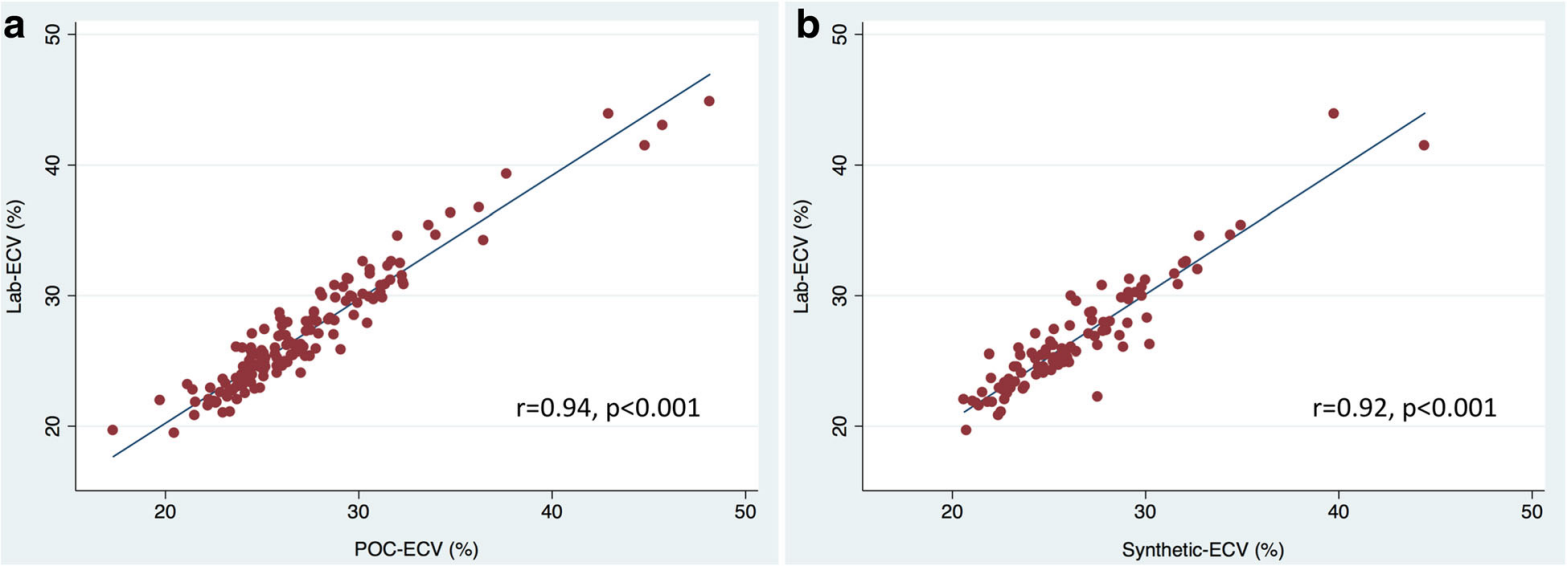
Linear correlation between laboratory and point-of-care (POC) derived extracellular volume (ECV) (**a**) and laboratory and synthetic derived ECV (**b**)

**Fig. 3:**
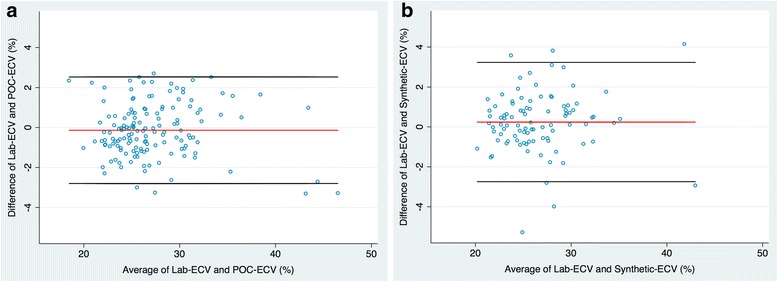
Bland-Altman plots of the mean differences between laboratory and point-of-care (POC) derived extracellular volume (ECV) (**a**) and laboratory and synthetic derived ECV (**b**). For each parameter in (**a**) and (**b**), the average of measurements from both techniques is plotted on the x-axis and the difference between techniques is plotted on the y-axis. The solid red horizontal line plots the mean difference and the solid black lines indicated the limits of agreement (differences from the mean of 1.96 SDs) for each parameter

POC analysis was successfully performed in 63% (*n* = 77) of subjects on the first attempt, in 70% (*n* = 86) within two attempts, and in 91% (*n* = 112) within three attempts. The mean duration for POC analysis was 82 ± 9 s (range 70–105 s). Mean POC-Hct was 0.395 ± 0.044 with no significant difference between POC-Hct and Lab-Hct (*p* = 0.149). Mean POC-ECV was 27.3 ± 4.8% with no significant difference between POC-ECV and Lab-ECV (*p* = 0.106). There was good correlation between laboratory and POC measures of hematocrit (*r* = 0.81, *p* < 0.001) and excellent correlation between laboratory and POC measures of ECV (*r* = 0.94, *p* < 0.001), Fig. 2a. On Bland-Altman analysis, there was minimal bias for POC-ECV in comparison to Lab-ECV with modest precision (bias = − 0.18%, 95%CI [− 2.85, 2.49], confidence limit 5.34%), Fig. 3a.

Synthetic-Hct was derived from blood pool T1 values in all subjects imaged at 1.5 T (*n* = 74). There was no statistically significant difference between Lab-Hct and synthetic-Hct (0.392 ± 0.042 vs. 0.398 ± 0.031, *p* = 0.074) or Lab-ECV and synthetic-ECV (26.7 ± 4.4% vs. 26.5 ± 4.3%, *p* = 0.084) in the subgroup imaged at 1.5 T. There was moderate correlation between laboratory and synthetic measures of hematocrit (*r* = 0.60, *p* < 0.001) and excellent correlation between laboratory and synthetic measures of ECV (*r* = 0.92, p < 0.001), Fig. 2b. On Bland-Altman analysis, there was slightly larger bias for synthetic-ECV in comparison to Lab-ECV and slightly wider limits of agreement (bias = 0.23%, 95%CI [− 2.82, 3.27], confidence limit 6.09%), Fig. 3b.

### Sensitivity analysis

There was no statistically significant difference between POC-ECV and Lab-ECV when analysis was restricted to different subgroups, including subjects imaged at 1.5 T (*n* = 74, *p* = 0.410), imaged at 3 T (*n* = 49, *p* = 0.120), females (*n* = 71, *p* = 0.458), males (*n* = 52, *p* = 0.087), healthy subjects (*n* = 19, *p* = 0.376), and patients (*n* = 104, *p* = 0.164). However, there was a significant difference between POC-ECV and Lab-ECV when analysis was restricted to subjects with anemia (*n* = 31, *p* = 0.009).

There was no significant difference between synthetic-ECV and Lab-ECV when analysis was restricted to males (*n* = 22, *p* = 0.249) and healthy subjects (*n* = 12, *p* = 0.182). However, there were significant differences between synthetic-ECV and Lab-ECV when analysis was restricted to females (*n* = 52, *p* = 0.008), patients (*n* = 62, *p* = 0.018), and subjects with anemia (*n* = 22, *p* = 0.001), suggesting that synthetic-ECV may be less robust compared to POC-ECV.

### Diagnostic performance

ROC curves were used to evaluate the diagnostic performance of POC-ECV and synthetic-ECV in comparison to Lab-ECV as the reference standard using a cut-off of Lab-ECV ≥30% as abnormal. POC-ECV had good diagnostic test performance for discriminating Lab-ECV ≥30% (sensitivity 85%, specificity 96%, accuracy 94%, and area under the curve (AUC) 0.902, 95%CI[0.829, 0.976]). Synthetic-ECV had moderate diagnostic test performance (sensitivity 71%, specificity 98%, accuracy 93%, and AUC 0.849, 95%CI[0.725, 0.973]). There was no significant difference in AUC between the two noninvasive measures when analysis was restricted to studies performed at 1.5 T (*p* = 0.629).

### Test-retest and inter-observer agreement

Excellent test-retest (ICC 0.951, 95%CI[0.905, 0.977]) and inter-observer agreement (ICC 0.900, 95%CI[0.719, 0.965]) were demonstrated for POC-Hct. Excellent test-retest (ICC 0.994, 95%CI[0.987, 0.997]) and inter-observer agreement (ICC 0.974, 95%CI[0.929, 0.991]) were demonstrated for POC-ECV.

### Comparison of POC devices

In the group of subjects who underwent parallel hematocrit assessment with two POC devices and laboratory determination, there was no significant difference in hematocrit assessed using the Pronto^®^ device (0.408 ± 0.045) compared to the Pronto-7^®^ device (0.392 ± 0.045, *p* = 0.135) and compared to Lab-Hct (0.405 ± 0.044, *p* = 0.830). Similarly, there was no significant difference in ECV derived using hematocrit from the Pronto^®^ POC device (24.3 ± 3.0%) compared to ECV derived using hematocrit from the Pronto-7^®^ POC device (25.0 ± 2.9%, *p* = 0.124) and compared to ECV derived from Lab-Hct (24.3 ± 2.2%, *p* = 0.959).

## Discussion

The results of this prospective study demonstrate that accurate and reproducible CMR ECV values can be calculated with input of hematocrit measured using a noninvasive POC device, potentially eliminating the need for blood collection by venipuncture for the purpose of ECV analysis. POC-ECV values correlate strongly with conventionally calculated ECV using laboratory hematocrit as the reference standard, with good diagnostic test performance, minimal bias and good agreement with Lab-ECV in both genders. Synthetic-ECV has slightly larger bias, wider limits of agreement and is less robust on sensitivity analyses when compared to POC-ECV.

There is growing interest in quantifying myocardial ECV as a marker of diffuse interstitial myocardial changes including fibrosis [22]. Myocardial ECV correlates with histologic measures of diffuse myocardial fibrosis [23, 24], and is independently associated with adverse outcomes including mortality [4]. However, clinical implementation of myocardial ECV may be limited by the requirement for timely hematocrit assessment [25]. Hematocrit is conventionally assessed by a peripheral blood sample obtained by venipuncture which is often inconvenient and uncomfortable for the patient. Several methods of noninvasive hematocrit evaluation have recently become commercially available which could be implemented to streamline CMR ECV evaluation.

Noninvasive POC devices provide rapid on the spot measures of hemoglobin without the need for blood sampling [15, 26]. Most noninvasive POC devices rely on a spectrophotometer finger probe sensor to analyze light absorption characteristics of different hemoglobin species [27]. Prior studies utilizing earlier POC devices reported conflicting results [28, 29]. However, comparison of earlier results to current POC device performance is limited given ongoing software and hardware revisions [30]. More recent generations of POC devices (including the device used in this study), have been evaluated in subjects in a variety of clinical settings [9, 31, 32] and in healthy volunteers [33, 34], with good correlation between POC hemoglobin and laboratory reference measures. A recent large meta-analysis evaluating the accuracy and precision of current POC devices against central laboratory hemoglobin measurements reported a pooled random-effects mean difference of POC minus central laboratory hemoglobin values of 0.10 ± 1.37 g/dL (limits of agreement − 2.59 to 2.80 g/dL) [15]. Similarly, the results of our study demonstrate a small mean difference between POC-ECV and Lab-ECV of − 0.18% with relatively narrow limits of agreement (− 2.85 to 2.49%). Notably, previous publications have not evaluated the diagnostic performance of POC derived ECV values, which is an important strength of the current study.

We evaluated POC device performance in the calculation of myocardial ECV in a relatively diverse cohort of subjects, and therefore results are applicable in a relatively broad range of clinical and research settings. POC analysis was quick, with most measurements acquired with a single analysis attempt. This noninvasive measure of hematocrit was easily incorporated into the CMR workflow and can be implemented in myocardial ECV calculation with the following caveats. POC analysis was not possible in a small subset of subjects, including subjects with applied gel nail polish, marked hand tremor and poor peripheral perfusion, which are recognized limitations of POC device analysis [28, 35]. Additionally, in the subset of subjects with anemia, there was a significant difference between laboratory and POC ECV values. This may be explained by the fact that low hemoglobin levels have been shown to impair perfusion analysis [36]. Given this finding, POC derived hematocrit is not recommended for CMR ECV calculation in subjects with anemia. This finding can be further explored in future studies that include a larger number of subjects with anemia.

A few recent studies have reported synthetic derivation of hematocrit and ECV based on the linear relationship between hematocrit and non-contrast R1 of blood [11, 13]. Automated inline derivation of synthetic hematocrit with subsequent calculation of myocardial ECV could potentially improve workflow, eliminating the need to acquire an invasive hematocrit value at the time of CMR. Treibel et al. demonstrated moderate correlation between synthetic and laboratory hematocrit (r^2^ = 0.51–0.45, *p* < 0.001) and strong correlation between synthetic and laboratory derived ECV (r^2^ = 0.97, *p* < 0.001) [11]. Our study similarly demonstrates excellent correlation between synthetic and laboratory ECV, although the mean difference between synthetic-ECV and Lab-ECV was slightly greater and the limits of agreement were slightly wider when compared to POC-ECV. A recent study investigating synthetic ECV in a pediatric population reported 41 (25%) false negatives and 4 (2%) false positives for mid-septal synthetic ECV using a published model and cut-off value of 28.5% [13]. The authors conclude that use of synthetic hematocrit for the calculation of ECV results in miscategorization of individual patients. Similarly, the results of the current study demonstrate that synthetic-ECV values are less robust on sensitivity analysis compared to POC-ECV, with significant differences between laboratory and synthetic ECV values when restricted to females, patients and those with anemia. Synthetic hematocrit and ECV values are affected by factors that affect non-contrast blood pool T1 values, such as changes in oxygen and iron concentration and body temperature [37–40]. There are also important practical limitations to synthetic analysis, as formulas to calculate synthetic hematocrit should be derived at each institution in a large sample of patients imaged on each individual scanner and with each specific acquisition scheme to which the results will be applied [12]. We conclude that synthetic-ECV is less robust compared to POC-ECV, and requires further evaluation prior to clinical implementation.

Our study has a number of limitations. First, the non-invasive POC device used in this study became commercially unavailable after this study was completed. However, multiple other non-invasive hemoglobin monitoring devices are currently available, including Pronto^®^ Pulse CO-Oximeter^®^ (Masimo Personal Health), Radical-7^®^ Pulse CO-Oximeter^®^ (Masimo Personal Health), NBM-200 (OrSense, Petah-Tikva, Israel) and Haemospect^®^ (MRB Optical Systems GmbH & Co. KG, Wuppertal, Germany). Several prior studies have compared hemoglobin results from the Pronto-7^®^ device used in this study and NBM-200 device, with similar accuracy and bias reported between devices [26, 41–43]. A recent meta-analysis evaluating the accuracy of non-invasive hemoglobin monitoring devices reported that pooled mean differences and standard deviations were similar among three devices included in the analysis (Radical-7^®^, Pronto-7^®^, and NBM-200 devices) [15]. We demonstrate no significant difference in hematocrit or ECV assessed using the Pronto^®^ Pulse CO-Oximeter^®^ compared to the Pronto-7^®^ Pulse CO-Oximeter^®^ and to laboratory values. These results suggest that currently available non-invasive hemoglobin monitoring devices will most likely lead to similar findings as reported in this study. Second, diurnal and monthly within-subject fluctuations in hematocrit are a recognized phenomenon, estimated at 3% [44, 45], with additional variations in hematocrit values linked to season, age, hydration and physical activity [46–49]. Our study used a single laboratory hematocrit measure within 24 h of CMR as the reference standard. It is possible that slight fluctuations in hematocrit between the time of CMR, POC device acquisition and peripheral blood sampling for laboratory analysis could have influenced the results. The median interval between laboratory hematocrit analysis and CMR was only 1.3 h and therefore the impact of potential variations in hematocrit between analysis methods is likely minimal. Third, non-invasive hemoglobin measurements may vary between POC devices and settings. However, a recent meta-analysis has demonstrated good agreement in hemoglobin between various POC devices [15]. The presence of an arrhythmia, including atrial fibrillation, was not recorded at the time of CMR. Therefore, the accuracy of POC devices in the setting of an arrhythmia has not been evaluated. Finally, the sample size was modest and all data were acquired at a single institution. Further multi-center studies including a larger number of patients with anemia should be performed to validate these results.

## Conclusions

CMR ECV calculated with input of hematocrit measured using a noninvasive POC device is accurate and reproducible compared to conventionally calculated ECV using laboratory hematocrit, potentially overcoming an important barrier to clinical implementation of ECV measurements. Further evaluation of synthetic ECV is required prior to clinical implementation.

## Abbreviations

AUC: Area under the curve
bSSFP: Balanced steady-state free precession
CMR: Cardiovascular magnetic resonance
ECV: Extracellular volume
Hct: Hematocrit
ICC: Intra-class correlation coefficient
IQR: Interquartile range
Lab-ECV: Laboratory extracellular volume
Lab-Hct: Laboratory hematocrit
LGE: Late gadolinium enhancement
LV: Left ventricle/left ventricular
MOLLI: MOdified Look-Locker Inversion
POC: Point-of-care
POC-ECV: Point-of-care extracellular volume
POC-Hct: Point-of-care hematocrit
ROC: Receiver operating curve
Synthetic-ECV: Synthetic extracellular volume
Synthetic-Hct: Synthetic hematocrit
